# Cinobufacini Injection Inhibits the Proliferation of Triple-Negative Breast Cancer Through the Pin1–TAZ Signaling Pathway

**DOI:** 10.3389/fphar.2022.797873

**Published:** 2022-04-05

**Authors:** Lu Kong, Xu Liu, Bing Yu, Ye Yuan, Qianru Zhao, Yuru Chen, Bin Qu, Xue Du, Xiaoxuan Tian, Rui Shao, Yu Wang

**Affiliations:** ^1^ School of Integrative Medicine, Tianjin University of Traditional Chinese Medicine, Tianjin, China; ^2^ Laboratory of Pharmacology of TCM Formulae Co-Constructed by the Province-Ministry, Tianjin University of Traditional Chinese Medicine, Tianjin, China; ^3^ State Key Laboratory of Component-Based Chinese Medicine, Tianjin University of Traditional Chinese Medicine, Tianjin, China; ^4^ Tianjin Central Hospital of Gynecology Obstetrics, Tianjin, China; ^5^ Biophysics, Center for Integrative Physiology and Molecular Medicine (CIPMM), School of Medicine, Saarland University, Homburg, Germany; ^6^ INM-Leibniz Institute for New Materials, Saarbruecken, Germany; ^7^ Tianjin Union Medical Centre, Tianjin, China

**Keywords:** cinobufacini injection, triple-negative breast cancer, TAZ, Pin1, proliferation

## Abstract

Triple-negative breast cancer (TNBC) is an aggressive subtype of breast cancer (BC), which is characterized by the total absence of human epidermal growth factor receptor 2 (HER2), progesterone receptor (PR), and estrogen receptor (ER) expression. Cinobufacini injection (CI) is the aqueous extract from the dry skin of *Bufo gargarizans*, which is broadly used for the treatment of malignant tumors. However, the potential mechanism of CI against TNBC has not been fully revealed. In this study, we found that CI inhibited the proliferation of MDA-MB-231 and 4T1 cells in a time- and dose-dependent manner. RNA-seq data showed that downregulated and upregulated genes were mainly enriched in biological processes related to tumor cell proliferation, including cell cycle arrest and regulation of apoptosis signaling pathways. Indeed, after CI treatment, the protein level of CDK1 and Bcl-2/Bax decreased, indicating that CI induced the cell cycle of MDA-MB-231 arrest in the G2/M phase and increased the rate of apoptosis. Meanwhile, CI significantly inhibited the growth of tumor *in vivo*, and RNA-seq data showed that the TAZ signaling pathway played a vital role after CI treatment. Both immunohistochemistry and Western blot analysis confirmed the downregulation of Pin1 and TAZ, caused by CI treatment. Furthermore, the bioinformatics analysis indicated that Pin1 and TAZ were indeed elevated in TNBC patients, with poor staging, classification, and patient survival rate. In conclusion, CI effectively inhibited the proliferation of TNBC *in vitro* and *in vivo* and induced their apoptosis and cycle arrest through the Pin1–TAZ pathway.

## Introduction

Breast cancer is a female malignant tumor and the most common in the world and the leading cause of cancer death (11.6% of the total cancer deaths). Breast cancer seriously endangers human health, and there were more than two million new cases and more than 620,000 deaths, in 2018 (Bray et al., 2018; Ahmad, 2019). Triple-negative breast cancer is usually defined as a type of breast cancer that lacks HER2, PR, and ER (Ahmad, 2019; Lee et al., 2019), which has the characteristics of low age of onset, strong invasiveness, poor prognosis, high metastasis rate, recurrence rate, and mortality rate (Won and Spruck, 2020). Current treatments for BC include surgical treatment, adjuvant chemotherapy, endocrine therapy, molecular-targeted therapy, and traditional Chinese medicine (TCM) treatment. (Chaudary et al., 2018). Due to the negative expression of HER2, ER, and PR, TNBC is not sensitive to endocrine therapy and targeted therapy, and patients usually relapse within 5 years after surgery, and the overall prognosis is poor (Vagia et al., 2020). Thus, there is an urgent demand for more efficacious and less toxic drugs to treat TNBC.

The peptidylprolyl cis–trans isomerase NIMA-interacting 1 (Pin1) has been considered to be a novel biomarker for the stratification of TNBC patients for treatment, in order to improve the management of poor outcome of cancer. As a prolyl isomerase, Pin1 has the function of catalyzing the cis–trans isomerization of the phosphorylated serine/threonine–proline (pSer/Thr-Pro) motif (Chuang et al., 2021). This Pin1-dependent isomerization leads to changes in function and thus the conformation, of many key proteins, which play key roles in many signaling pathways implicated in cancer, including β-catenin, ER-α, NFκB, Stat3, cyclin D1, Notch, and AKT (Chen et al., 2018). Mounting evidence has revealed that Pin1 is usually highly expressed in TNBC and closely related to the clinical stage, distant metastasis, and prognosis of TNBC patients (Knowlson et al., 2020). Also, some studies have shown that knocking out Pin1 can cause cell death in TNBC, but the opposite is true in normal cells (Karna et al., 2019). Hence, Pin1 is regarded as an intriguing target for TNBC therapy.

Cinobufacini, an extract of dried toad skin, is a traditional Chinese antitumor medicine with a wide range of clinical applications (Lu et al., 2008; Qi et al., 2010a; Mao et al., 2020). Cinobufacini is available in a variety of dosage forms; cinobufacini injection (CI) is a water-soluble preparation made from the skin of *Bufo gargarizans*. In the past 10 years, CI has long been used alone or combined with chemotherapy drugs in the synthesized management of cancers, such as breast cancer, lung cancer, hepatocellular carcinoma, prostate cancer, and gallbladder cancer, as a long-term supplement and alternative therapy (Qin et al., 2008; Jiang et al., 2010; Dong et al., 2016; Chen et al., 2018). In addition, CI was found to be able to regulate the process of apoptosis and cycle arrest in human breast cancer (MDA-MB-231 and MCF-7 cells), human T-cell leukemia (Jurkat T cells), and human lung cancer (A-549 cells) (Dong et al., 2016; Li et al., 2021). However, the molecular mechanism of CI against TNBC still remains unclear.

In our study, we explored the effect of CI on TNBC and investigated its underlying molecular mechanism. Combined with bioinformatics technologies, our study elucidated for the first time that CI played its anticancer roles on TNBC by downregulating the Pin1–TAZ pathway. This not only highlights the importance of the Pin1–TAZ pathway in the progression of TNBC but also provides the developing treatment strategies for TNBC.

## Materials and Methods

### Cell Culture and Reagents

CI (Cat. Z34020273, Lot.200,505-2) was obtained from Anhui China Resources Jinchan Pharmaceutical Co., Ltd. All the cell lines were purchased from the American Type Culture Collection and cultured in our laboratory. All of them were cultured in RPMI-1640 (Gibco) and Dulbecco’s modified Eagle medium (Gibco) containing 10% fetal bovine serum (Vian-Saga) and 1% antibiotics (HyClone) under the humidified condition with 5% CO_2_, at 37°C.

### Cell Viability Assay

Cell viability was detected using the CCK8 assay. Briefly, MDA-MB-231 and 4T1 cells (2–4×10^3^ cells/well) were seeded in 96-well plates and incubated for 24 h. Then, the cells were treated with various dilutions of CI. After treatment for 24, 48, and 72 h, a 10 μL CCK8 solution (Dojindo, Japan) was added to each well and incubated for additional 2–4 h at 37°C. The absorbance was measured using ELISA Reader (TECAN) for living cells at 450 nm. The analysis was carried out in three replicates.

### Transfection Assay

The siRNA sequences targeting Pin1 (20 µM) were as follows: Pin1 siRNA1, 5′-CCG​UGU​UCA​CGG​AUU​CCG​GCA​UCC​A-3′, Pin1 siRNA2, 5′-GCCCUGGAGC UG AUCAACGGCUACA-3′. The transfection was performed using Lipofectamine^®^ 2000 (Invitrogen; Thermo Fisher Scientific, Inc.). According to the manufacturer’s protocol, the transfection medium was replaced with a complete medium 6 h after transfection at 37°C, after which the cells were incubated for the indicated times. All treatments were started at 48 h after transfection.

### Colony Formation Assay

For performing colony formation assays, TNBC cells were seeded into six-well plates and cultured overnight. Then, the cells were treated with different concentrations of CI. On the 14th day, the colonies were fixed with 4% paraformaldehyde hydride and stained with 0.1% crystal violet. Count the number of colonies in the specified time period.

### Cell Apoptosis Analysis

The cells treated with different concentrations of CI for 12 and 24 h were harvested. Each sample was added with Annexin V-FITC and PI staining reagents after cells were washed with binding buffer. The samples were incubated for 15 min at room temperature away from light. Each sample was added with 300 μL binding buffer, and the samples were detected by flow cytometry.

### Cell Cycle Analysis

The cells treated with different concentrations of CI for 12 and 24 h were harvested. Then, the cells were washed with phosphate buffer saline (PBS) and were fixed with 75% ethanol for one night at −20°C. Discard ethanol and cells were washed with PBS. Each sample was added with RNase and incubated at 37°C for 30 min. Each sample was added with PI, PBS and incubated at 37°C for 30 min away from light. Each sample was added with PBS and was detected by flow cytometry.

### RNA Sequencing

The samples were prepared with MDA-MB-231 cells and 4T1 tumor treated with CI. The control group was administered with normal saline. The samples were collected and total RNA was extracted. After the enriched mRNA was reverse transcribed into cDNA, cDNA library was established, and Illumina sequencing was performed. Differentially expressed genes were subjected to KEGG pathway enrichment analysis on the Novomagic cloud platform (https://magic.novogene.com), and the WeChat online mapping website (https://www.bioinformatics.com.cn), and Cytoscape software (version 3.8.2) was used for mapping.

### Western Blot Assay

For cells and tumors, the protein was extracted from the cell lysate with RIPA buffer (Beyotime, China) supplemented with inhibitor cocktail (Roche) and PMSF (Beyotime, China) after centrifuging twice. Then, the protein quantification was performed using the BCA method. The protein was transferred to PVDF membranes after being separated by SDS-PAGE. Subsequently, the membrane was blocked in TBST containing 5% skimmed milk for 2 h. The PVDF membrane (GE Healthcare Life science, Germany) was incubated at 4°C overnight with the flowing antibodies: Bcl-2(CST, 4223S), Bax (CST, 2272S), CDK1 (Abcam, Ab133327), TAZ (Abcam, Ab224239), Pin1(Abcam, Ab191271), β-actin (ZSGB-Bio, 7A-09). The next day, the membrane was washed three times with TBST (0.05% Tween), and then the secondary HRP-conjugated anti-mouse/anti-rabbit antibody was incubated with the membrane for 1 h at room temperature after washing three times with TBST. Finally, the protein bands were detected using the enhanced chemiluminescence (ECL) detection reagents, and the band intensity was quantified using ImageJ software.

### Ectopic Subcutaneous Tumor Model

4T1 cells (3 × 10^6^cells/mL) were inoculated in the armpits of mice and the nude mice xenograft model bearing human MDA-MB-231 cells (2 × 10^7^cells/mL). After the mice grew a tumor, the mice were randomly divided into four groups, and received CI (low dose: 0.25 g/kg; high dose: 0.5 g/kg) or DOX (5 mg/kg). The long and short diameters of the tumor tissue were measured using a Vernier caliper, and the weight of the mice was recorded every 5 days. The mice were killed to collect major target organs. Then, the tumor tissues were stripped and placed neatly and photographed. The tissue was fixed with 4% paraformaldehyde for immunohistochemistry experiments, and a part of the tumor tissue was kept frozen for other experiments. All the animal experiments were conducted under protocols approved by the Animal Care and Use Committee of Tianjin University of Traditional Chinese Medicine.

### Immunohistochemistry Assay

Paraffin-embedded tissue sections were dewaxed with xylene, rehydrated with decreasing concentrations of ethanol, and washed with water. Following high-pressure antigen retrieval with 10 mM citrate buffer, the slices were soaked in 3% hydrogen peroxide to inactivate the exogenous peroxidase activity, and then blocked with the goat serum working solution. Next, the sections were incubated at 4°C with the primary antibody Pin1 (Abcam, Ab191271), TAZ (Abcam, Ab224239), and Ki67 (Abcam, Ab15580) overnight, and rinsed with PBS. Then, the sections were incubated with the horseradish peroxidase (HRP)-labeled goat anti-rabbit secondary antibody for 30 min at 37°C. Afterward, the sections were developed with diaminobenzidine (DAB) for the appropriate time, washed under running water, and counterstained with hematoxylin. Then, the sections were washed under running water and differentiated with hydrochloric acid–alcohol. After differentiation, the sections were dehydrated through gradient ethanol and xylene. Then, the stained sections were observed under a microscope after sealing with neutral resin glue.

### Bioinformatics Analysis

The Cancer Genome Atlas (TCGA: http://cancergenome.nih.gov/) data were analyzed to compare mRNA expression in normal tissues and tumor tissues and to compare the expression of Pin1 and TAZ in different types and stages of TNBC circumstances and finally to study the relationship between Pin1, TAZ, and patient survival rate.

### Statistical Analysis

All values are shown as mean ± SD. Statistical analysis was performed with GraphPad Prism software. Differences between groups were examined for statistical significance with one-way ANOVA. *p* < 0.05 was considered a statistically significant difference.

## Results

### CI Inhibits the Viability of TNBC Cells

**FIGURE 1:**
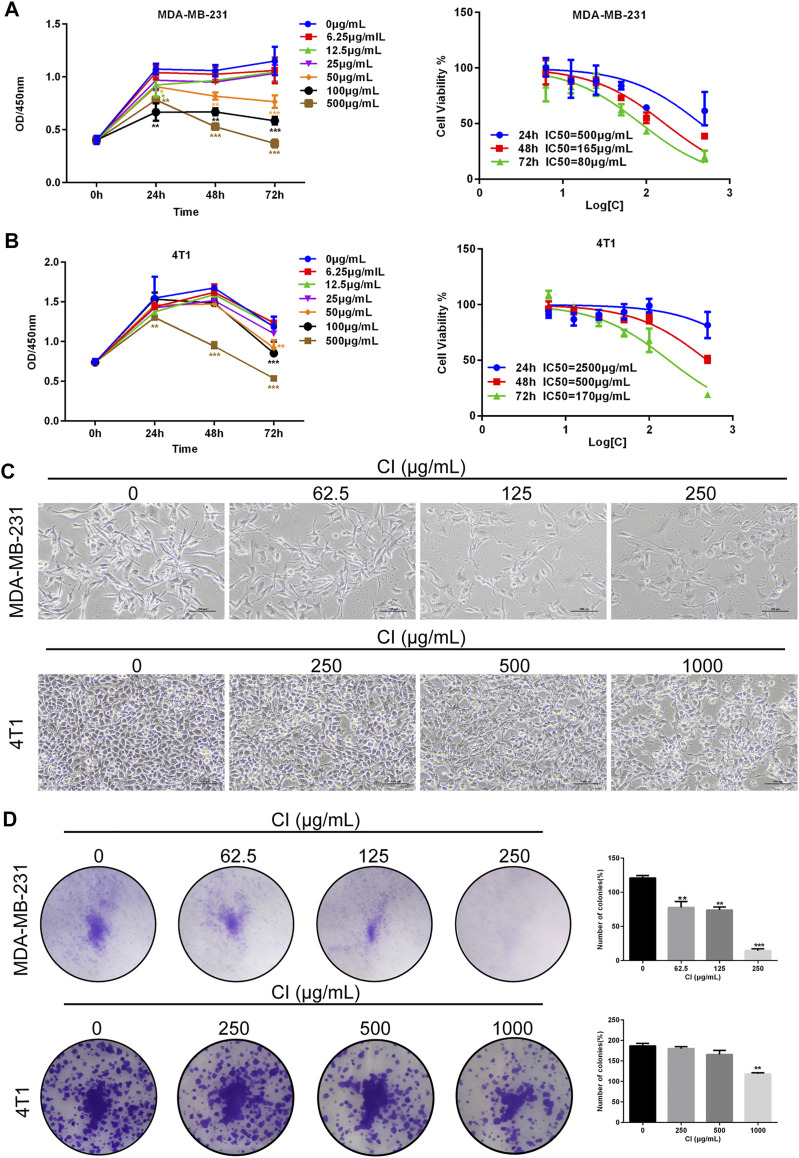
CI reduces cell viability of TNBC cells. **(A)** MDA-MB-231 cells was treated with CI (0, 6.25, 12.5, 25, 50, 100, and 500 μg/ml) for 24, 48, and 72 h, and cell viability was determined by the CCK8 assay. **(B)** 4T1 cells was treated with CI (0, 6.25, 12.5, 25, 50, 100, and 500 μg/ml) for 24, 48, and 72 h, and cell viability was determined by theCCK8 assay. **(C)** Morphological changes of MDA-MB-231 and 4T1 cells treated with CI (0, 62.5, 125, and 250 μg/ml or 0, 250, 500, and 1000 μg/ml). **(D)** Colony formation assay of MDA-MB-231 and 4T1 cells exposed to CI (0, 62.5, 125, and 250 μg/ml or 0, 250, 500, and 1000 μg/ml) for 14 days. The colony numbers (>50 cells/colony) are calculated manually. *p < 0.05, **p < 0.01, ***p < 0.001, and ****p < 0.0001 *vs.* control.

The cell viability of MDA-MB-231 and 4T1 cells after cells were treated with various dilutions of CI (6.25, 12.5, 25, 50, 100, and 500 μg/ml) for 24, 48, and 72 h, measured using the CCK8 assay, decreased significantly in a time and dose-dependent manner (Figures 1A, B). Meanwhile, under the microscope, we observed that compared with controls, CI treatment significantly declined the cell number and inhibited the colony formation of MDA-MB-231 and 4T1 cells in a dose-dependent manner (Figures 1C, D).

### Effects of CI on Cell Cycle and Apoptosis of MDA-MB-231 Cells

**FIGURE 2:**
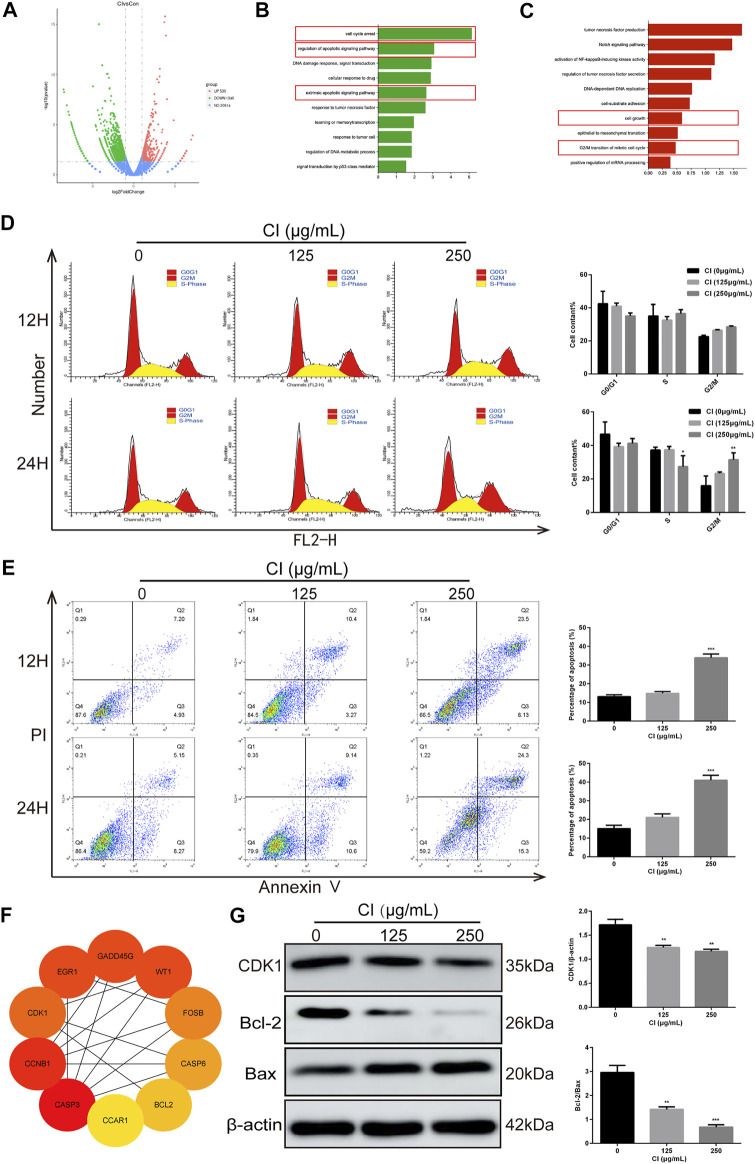
Analysis of CI (125 μg/ml)-regulated gene expression in MDA-MB-231 cells. **(A)** Upregulated and downregulated genes in MDA-MB-231 cells after CI treatment. **(B)** Genes generated from RNA-seq analysis that were annotated revealing key biological processes were upregulated. **(C)** Genes generated from RNA-seq analysis that were annotated revealing key biological processes were downregulated. **(D)** Cell cycle phases of MDA-MB-231 cell exposed to CI (0, 125, and 250 μg/ml) for 12 and 24 h were analyzed by flow cytometry. The cell cycle phase distribution is indicated as mean ± SD (*n* = 3). **(E)** Stained MDA-MB-231 cells exposed to CI (0, 125, and 250 μg/ml) for 12 and 24 h. Apoptotic cell proportions are expressed as mean ± SD (*n* = 3). **(F)** Top 10 genes related to tumor, cell cycle, and apoptosis. **(G)** Levels of expression of CDK1, Bax, and Bcl-2 were determined using Western blot assessment. *p < 0.05, **p < 0.01, ***p < 0.001 *vs.* control.

To determine the speculative target genes involved in the antitumor effect of CI, we used RNA sequencing analysis to detect the reactivity of the entire genome to CI in MDA-MB-231 cells. After 24 h of CI treatment with a concentration of 125 μg/ml, 530 genes were upregulated, and 1,340 genes were downregulated (*p* value < 0.05, |log_2_FoldChange| > 1) (Figure 2A). In order to further explore the molecular mechanism of the anti-proliferation effect of CI, enrichment analysis of the biological processes was established and showed that both of the up- and downregulated genes were mainly enriched in the biological processes related to tumor cell proliferation, including cell cycle arrest, regulation of apoptotic signaling pathway, apoptotic signaling pathway, cell growth, and G2/M transition of mitotic cell cycle (Figures 2B, C).

We further confirmed the role of CI on the progression of cell cycle and apoptosis in MDA-MB-231 cells *in vitro*. The results of flow cytometry showed that CI induced MDA-MB-231 cell cycle arrest in the G2/M phase (Figure 2D) and after treatment with CI for 12 and 24 h, the percentage of apoptotic cells significantly increased (Figure 2E). To investigate the mechanism behind CI-induced cell cycle arrest and apoptosis, we employed PPI (protein–protein interaction) to identify the top 10 genes related to tumor, cell cycle, and apoptosis, including CDK1 and Bcl2. (Figure 2F), which were verified using the Western blot assay (Figure 2G). All these data indicated that CI regulated the cell cycle and apoptosis in MDA-MB-231 cells.

### CI Inhibits Growth and Development in the TNBC Xenograft Mouse Model

**FIGURE 3:**
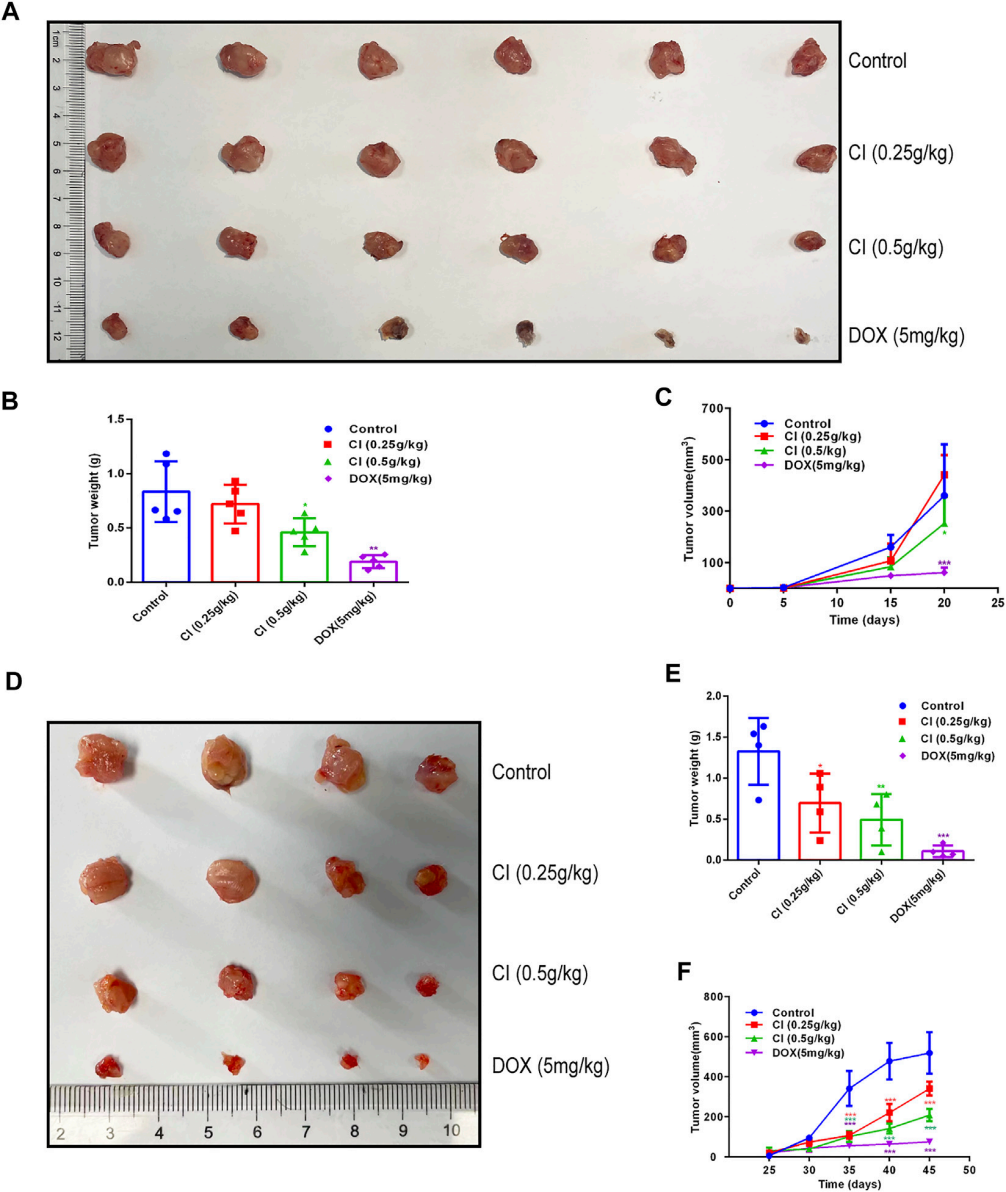
CI inhibits growth and development in the mouse model of TNBC (data of A, B, and C from 4T1 tumor-bearing mice and data of D, E, and F from MDA-MB-231 tumor-bearing mice). **(A)** Tumor tissues after sacrifice. **(B)** Tumor weight after CI treatment or DOX. **(C)** Tumor volume growth curve after CI treatment or DOX. **(D)** Tumor tissues after sacrifice. **(E)** Tumor weight after CI treatment or DOX. **(F)** Tumor volume growth curve after CI treatment or DOX. *p < 0.05, **p < 0.01, ***p < 0.001 *vs.* control.

We further investigated the effect of CI on a transplanted tumor growth produced by 4T1 and MDA-MB-231 cells. The growth of 4T1 tumor xenografts was inhibited significantly following the CI (Figures 3A–C). Likewise, the growth of MDA-MB-231 tumor xenografts was inhibited significantly (Figures 3D–F). To further evaluate the safety of CI, we performed the histopathological analysis. The results revealed by hematoxylin and H&E staining showed no significant injury in the main organs including the heart, liver, spleen, and kidney (Supplementary Figure S1C). Also, there is no significant change in body weight and organ coefficients (Supplementary Figures S1A, S1B). Notably, compared with the control group and the CI group, the tumor growth of the DOX group was more significantly inhibited, but the body weight and organ weight of the mice in this group decreased significantly, and the mice even died. The results of HE staining also showed that the organs of the mice in the DOX group were damaged.

### RNA Sequencing Revealed the Signal Pathways Altered by CI Treatment *In Vivo*

**FIGURE 4:**
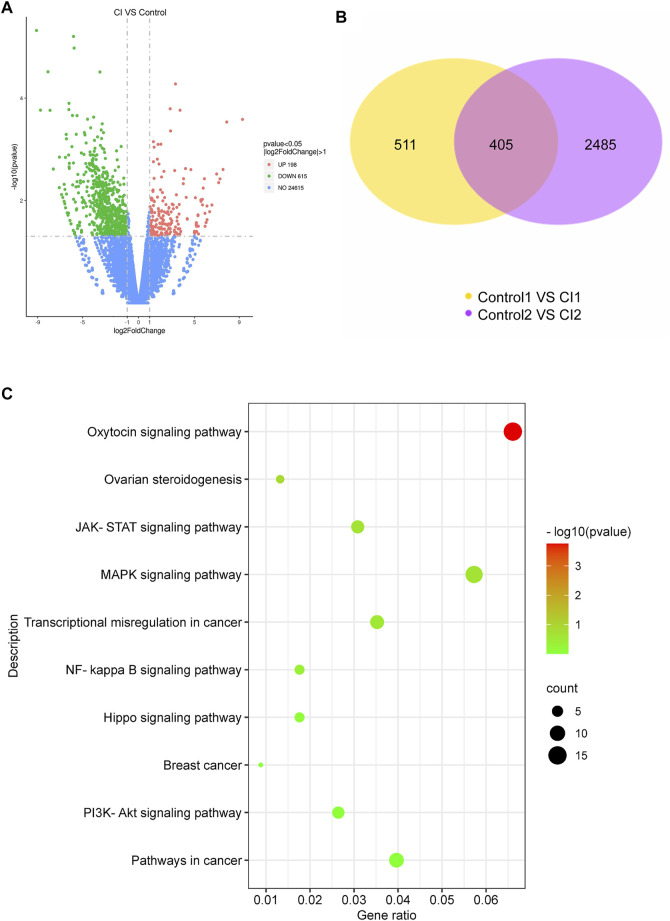
Analysis of CI (0.5 g/kg)-regulated gene expression in 4T1 tumor. **(A)** Upregulated genes and downregulated genes in 4T1 tumor. **(B)** Differentially expressed genes between CI and control. **(C)** Top 10 cancer-related pathways found by enrichment analysis of the KEGG pathway for differential genes.

To further investigate the effect of CI on TNBC gene expression, RNA-sequencing of tumor tissues was performed. After CI treatment, 198 genes were significantly up-regulated, and 615 genes were significantly downregulated in tumor tissues (*p* value < 0.05, |log_2_FoldChange| > 1) (Figure 4A). There are 511 differential expressed genes (DEGs) in the first comparison combination, 2,486 DEGs in the second comparison combination, and 405 DEGs shared by the two groups (Figure 4B). To further detect the potential mechanism of CI in TNBC, KEGG enrichment analyses were conducted. The top 10 cancer-related pathways in the KEGG pathway were shown (Figure 4C) which suggested that CI might play an anti-TNBC effect through multiple pathways.

### Pin1–TAZ Pathway Is Involved in the Anti-TNBC Effect of CI *In Vivo and In Vitro*

**FIGURE 5:**
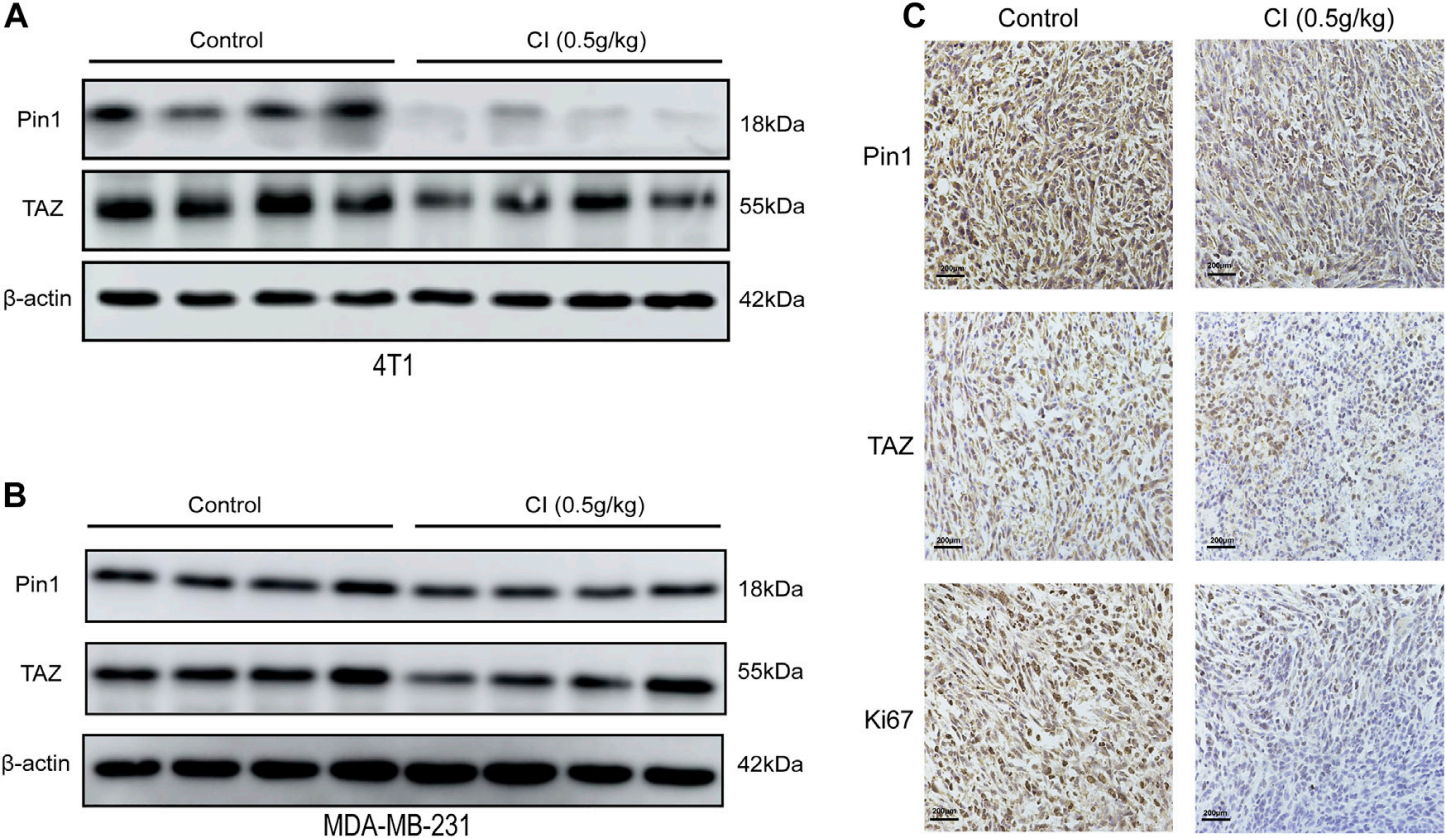
CI regulates the Pin1–TAZ signaling pathway. **(A)** Protein (Pin1 and TAZ) levels in tumor tissues of 4T1 tumor-bearing mice were detected by Western blot. **(B)** Protein (Pin1 and TAZ) levels in tumor tissues of MDA-MB-231 tumor-bearing mice were detected by Western blot. **(C)** Immunohistochemical analysis of paraffin-embedded 4T1 mice tumor tissue sections using antibody (Pin1, TAZ, Ki67). Scale bar stands for 200 μm.

**FIGURE 6:**
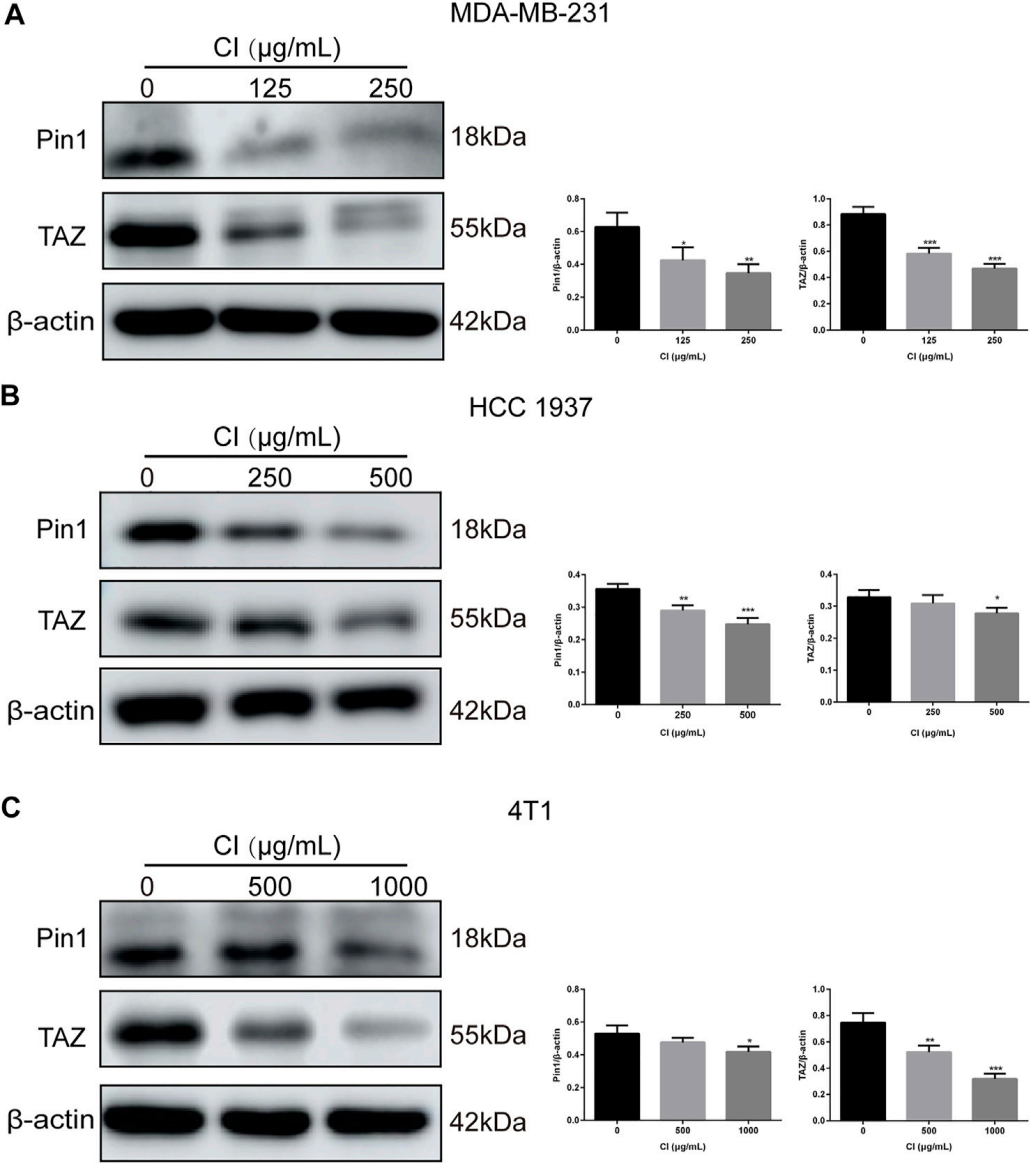
CI regulates the Pin1–TAZ signaling pathway in TNBC cells. **(A)** Levels of expression of Pin1 and TAZ in MDA-MB-231 cells treated with CI. **(B)** Levels of expression of Pin1 and TAZ in HCC 1937 cells treated with CI. **(C)** Levels of expression of Pin1 and TAZ in 4T1 cells treated with CI.

TAZ, one of the main downstream components of the Hippo pathway, plays a key role in the tumorigenic processes, *via* the transactivation of downstream genes (Deng et al., 2018; Kedan et al., 2018). In TNBC, Pin1 is the positive regulator of TAZ. In our RNA-seq data, both Pin1 and TAZ were indeed downregulated after CI treatment. To investigate the signal transduction pathway of CI inhibiting tumor growth, the expression levels of proteins that are involved in the Pin1–TAZ signaling pathway were determined in subcutaneous tumor models not only by the Western blot but also immunohistochemical analysis. Both assays showed that after CI treatment, the protein levels of Pin1 and TAZ decreased *in vivo* (Figure 5) and *in vitro* (Figure 6). Furthermore, knocking-down of Pin1 expression led to the down-expression of both Pin1 and TAZ (Supplementary Figure S3). These findings together suggest that CI may inhibit the tumor growth of TNBC by suppressing the Pin1–TAZ pathway.

### Expression of Pin1 and TAZ Is Closely Related to the Malignancy and Prognosis of TNBC

**FIGURE 7:**
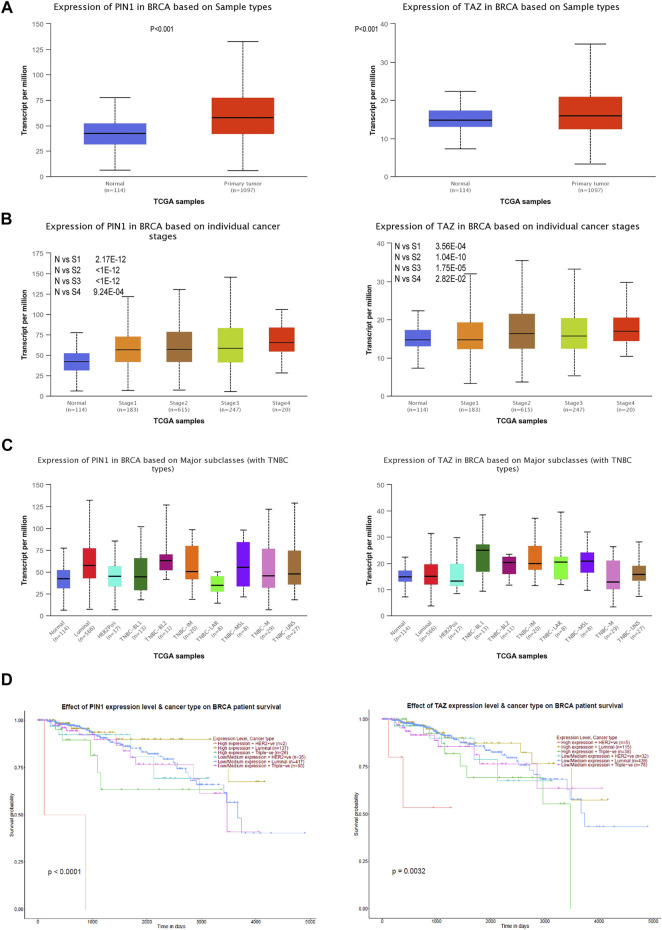
Pin1 and TAZ are highly expressed in TNBC. **(A)** Pin1 and TAZ are highly expressed in breast cancer. **(B)** Pin1 and TAZ are highly expressed in high-stage breast cancer. **(C)** Pin1 (N-vs-Luminal: 1.6 × 10^−12^, N-vs-HER2Pos: 0.18, N-vs-TNBC-BL1: 0.32, N-vs-TNBC-BL2: 0.0093, N-vs-TNBC-IM:0.0079, N-vs-TNBC-LAR: 0.85, N-vs-TNBC-MSL: 0.18, N-vs-TNBC-M:0.024, and N-vs-TNBC-UNS: 0.013.) and TAZ (N-vs -Luminal: 0.00012, N-vs-Her2Pos: 0.57, N-vs-TNBC-BL1: 0.007, N-vs-TNBC-BL2: 0.08, N-vs-TNBC-IM: 0.00068, N-vs-TNBC-LAR: 0.13, N-vs-TNBC-MSL: 0.08, N-vs-TNBC-M: 0.49, and N-vs-TNBC-UNS: 0.093) are highly expressed in TNBC. **(D)** Relationship between Pin1 and TAZ expression and patient survival in TNBC patients.

The Pin1 and TAZ difference analysis in TNBC were evaluated using UALCAN databases, and a prognosis analysis of Pin1 and TAZ based on Kaplan–Meier was performed (Chandrashekar et al., 2017). We found that Pin1 and TAZ were overexpressed in breast cancer than those in normal tissues (Figure 7A). Next, we analyzed the expression of Pin1 and TAZ in breast cancer subtypes and pathological stage. The data showed that compared with normal breast cancer, the expression levels of Pin1 and TAZ are increased in advanced breast cancer and are most significant in stage 4 (Figure 7B). Interestingly, compared with normal tissues and other breast cancer subtypes, Pin1, and TAZ are more highly expressed in TNBC (Figure 7C). Also, in TNBC patients, the higher the expression of Pin1 and TAZ indicates the lower survival rate of the patient (Figure 7D).

## Discussion

CI is a traditional Chinese antitumor drug with significant curative effects, which has been widely applied to a variety of solid tumors and leukemias, such as breast cancer, lung cancer, prostate cancer, hepatocellular carcinoma, and gallbladder cancer. More importantly, except for its anticancer effects, CI has a wealth of chemoprotection properties and lower toxicity (Ni et al., 2019). However, the molecular mechanism through which CI induces apoptosis and cycle arrest, and inhibits proliferation in TNBC cells has not been fully elucidated. In this present study, the CCK-8 experiments showed that CI had an anti-proliferative effect on MDA-MB-231 and 4T1 cells in a concentration-dependent manner. In addition, we used the mouse model of TNBC to observe the growth of tumor after CI treatment. Based on the results, we can conclude that CI can inhibit tumor proliferation *in vitro* and *in vivo* significantly. In addition, we also found that CI had no dose-limiting toxicities. The kidney, spleen, heart, and liver did not show typical pathological changes after CI treatment (Supplementary Figure S1).

Triggering apoptotic cell death is the main mechanism of most clinical chemotherapeutics in the treatment of TNBC. Thus, we studied the mechanisms by which CI inhibited the proliferation of TNBC cells and found that the proportion of apoptotic cells was significantly increased, and the proportion increased with the increase of the concentration after CI treatment. These results confirmed that CI exhibited an evident apoptosis-inducing effect on TNBC cells. The present study also revealed that CI induced the cell cycle arrest at the G2/M phase in MDA-MB-231 cells through down-regulating CDK1 expression. CDK1 binding with cyclin B is known as the main factor influencing the decision to enter mitosis. At the G2/M checkpoint, Pin1 can interact with the mitosis-related proteins Cdc25C and Wee1 to activate the cyclin B-CDK1 complex, thereby promoting the G2 phase of the cell cycle transition to the M phase (Cheng and Tse, 2018). The expression of Pin1 has been found to be related to the G2 phase of the cell cycle, and Pin1 can delay the entry of cells into mitosis, which indicates that Pin1 is involved in the regulation of cell cycle processes. Pin1 also binds to many cell cycle-regulatory proteins including Cdc25C, cyclin D1, p27, cyclin E, Myt1, and Wee1. In addition, Pin1 regulates cell apoptosis by directly regulating antiapoptotic proteins such as Bcl-2 and myeloid cell leukemia-1or pro-apoptotic proteins such as Bax and death-associated protein 6 (Daxx) or (Mcl-1) (Lu and Hunter, 2014). Compelling data suggest that Pin1 can treat human cancer and is targeted.

The transcriptional co-activator TAZ is the main downstream component of the Pin1 pathway (Hansen et al., 2015). TAZ participates in cell proliferation, drug resistance, and many other tumorigenic processes through the transactivation of downstream genes such as CTGF, Cyr61, and BMP4 in the nucleus (Han, 2019; Khanal et al., 2019; Mohajan et al., 2021). In addition, TAZ can affect the expression of Bcl-2, and thus participate in the regulation of cell apoptosis through the mitochondrial apoptosis pathway (Wang et al., 2016; Khanal et al., 2019). In the present study, we found that CI downregulated the expression of Pin1 and TAZ in TNBC cells *in vitro* and *in vivo*. In addition, the underlying mechanism of the CI regulating Pin1 pathway, either through inhibiting the PPIase activity of Pin1 or targeting the Pin1 WW domain to prevent the binding of Pin1 to its substrates, will be further studied.

CI is composed of multiple ingredients, in which bufogenin, bufalin, peptides, and indole alkaloids mainly contribute to its anticancer activity (Qi et al., 2011; Cheng et al., 2019). Both *in vitro* and *in vivo* assays indicate that cinobufacini and its active compounds (bufalin and bufogenin) repress tumorigenesis by inhibiting cell proliferation, inducing cell differentiation, inducing apoptosis, disrupting cell cycle, inhibiting cancer angiogenesis, reversing multidrug resistance, and regulating immune response (Qi et al., 2010b; Qi et al., 2011; Nakata et al., 2015). In addition, both RNA-seq assays from the TNBC cell line and xenografts have shown that CI plays an anti-TNBC effect by regulating multiple pathways, including PI3K-Akt, NF-κB, JAK-STAT, Pin1–TAZ, and other signaling pathways. Among them, the PI3K/AKT pathway is one of the active pathways involved in occurrence and development of TNBC and in the regulation, metabolism, cell proliferation, migration, and survival of TNBC cells (Costa et al., 2018; Deng et al., 2020; Liu et al., 2021). It has been extensively studied in a variety of cancer types, and many anti-TNBC drugs targeting PI3K-AKT are being studied (Khan et al., 2019). Assuredly, our data indicated that CI exerts an anti-TNBC effect through the PI3K-Akt pathway (Supplementary Figure S2). These analyses support the notion that CI exhibits anti-TNBC effects with multiple components acting on multiple targets. However, the active ingredients, which have an inhibitory effect on TNBC, remain unclear. Therefore, these studies encourage us to further conduct anti-TNBC research on cinobufacini and its active compounds.

## Conclusion

In summary, this study demonstrated that CI was able to induce apoptosis and cell cycle arrest via suppressing the Pin1–TAZ signaling pathway in TNBC cells *in vitro* and *in vivo*. CI may be proved to be a novel therapeutic strategy in the inhibition of carcinogenesis and progression of TNBC and provides the scientific basis for its clinical application as an anti-TNBC agent.

## Data Availability

The datasets presented in this study can be found in online repositories. The names of the repository/repositories and accession number(s) can be found at: BioProject, accession number PRJNA793163.

## Supplementary Material

Supplementary Figure S1 CI shows no obvious toxicity. **(A)** Mice weight growth curve after CI treatment or DOX. **(B)** Organ weight after CI treatment or DOX. **(C)** HE staining of major organs from mice treated with CI or DOX. Scale bar stands for 100 μm. **p* < 0.05, ***p* < 0.01, ****p* < 0.001, and *****p* < 0.0001 *vs.* control.

Supplementary Figure S2 Levels of expression of PI3K, AKT, and p-Akt were determined using the Western blot assessment.

Supplementary Figure S3 Transfection of Pin1 siRNA reduces Pin1 and TAZ protein levels. **(A)** Levels of expression of Pin1 and TAZ in MDA-MB-231 cells were determined using the Western blot assessment after transfection of Pin1 siRNA. **(B)** Levels of expression of Pin1 and TAZ in HCC 1937 cells were determined using the Western blot assessment after transfection of Pin1 siRNA.
