# Supplementary figures and images for: Cinobufacini Injection Inhibits the Proliferation of Triple-Negative Breast Cancer Through the Pin1–TAZ Signaling Pathway
Source: Front Pharmacol. 2022 Apr 5;13:797873. doi: 10.3389/fphar.2022.797873 (PMC9016199; doi:10.3389/fphar.2022.797873)

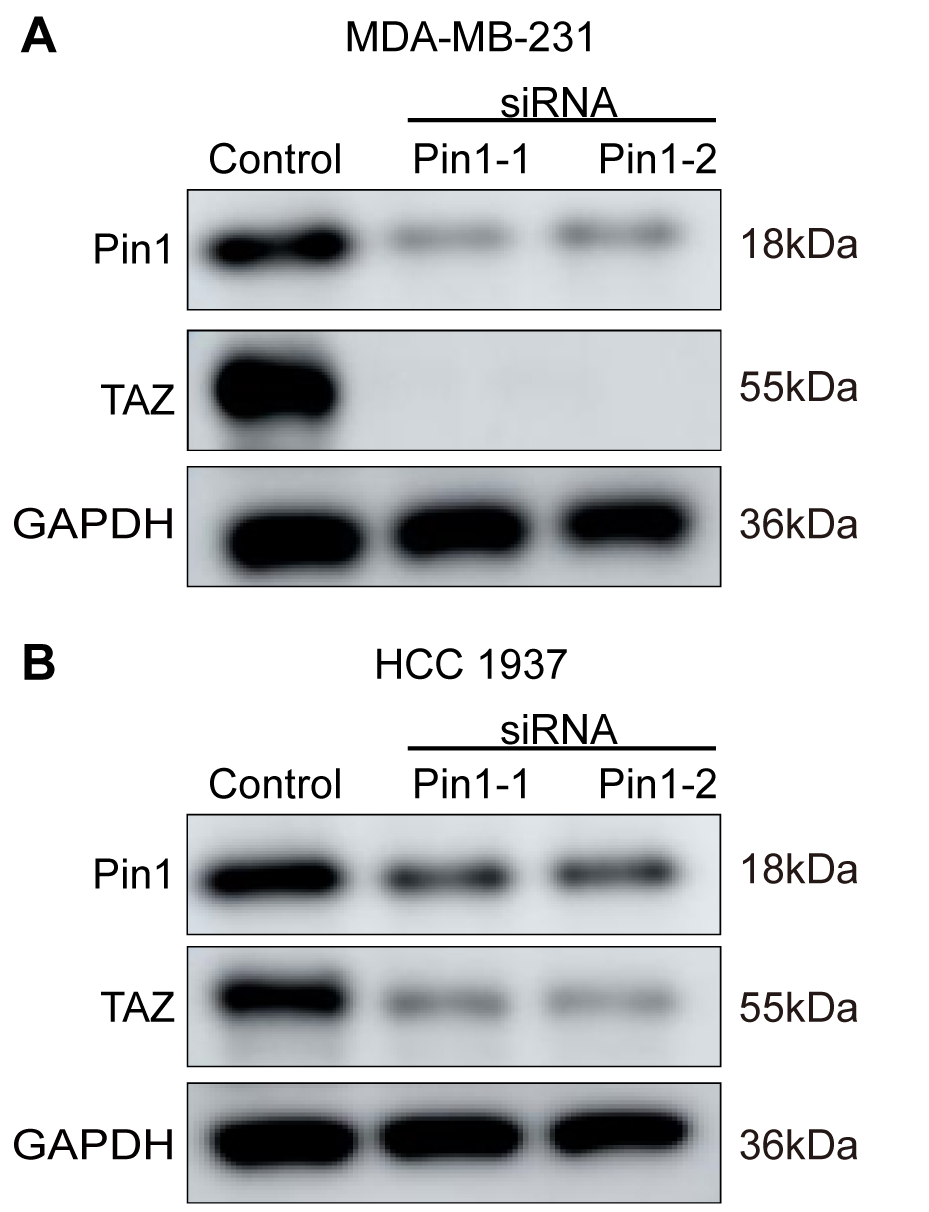

Supplement: Supplementary file 1 [file Image3.TIF]

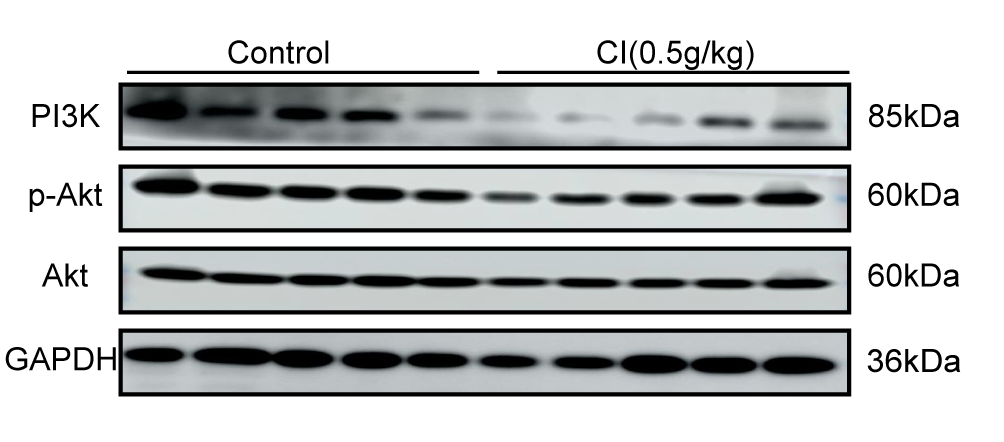

Supplement: Supplementary file 2 [file Image2.TIF]

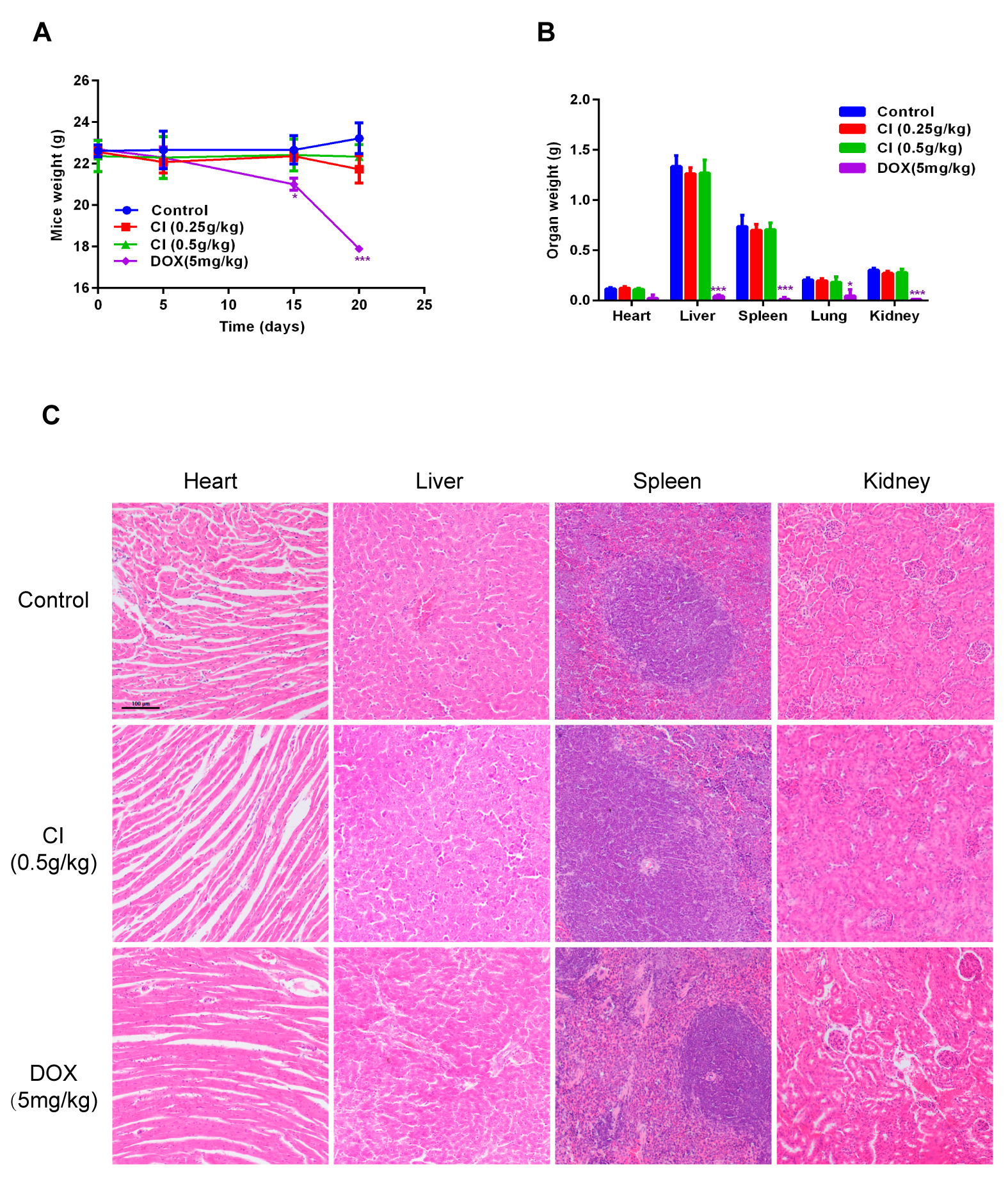

Supplement: Supplementary file 3 [file Image1.tif]
